# Midterm Results Comparing Perventricular Device Closure with Surgical Repair for Isolated Congenital Ventricular Septal Defects: A Systematic Review and Meta-Analysis

**DOI:** 10.31083/j.rcm2308262

**Published:** 2022-07-20

**Authors:** Juemin Yu, Rong Liufu, Ximeng Wang, Xiaobing Liu, Jian Zhuang

**Affiliations:** ^1^School of Medicine, South China University of Technology, 510641 Guangzhou, Guangdong, China; ^2^Department of Cardiovascular Surgery, Guangdong Cardiovascular Institute, Guangdong Provincial Key Laboratory of South China Structural Heart Disease, Guangdong Provincial People’s Hospital, Guangdong Academy of Medical Sciences, 510260 Guangzhou, Guangdong, China; ^3^Department of Cardiovascular Intensive Care Unit, Guangdong Cardiovascular Institute, Guangdong Provincial Key Laboratory of South China Structural Heart Disease, Guangdong Provincial People’s Hospital, Guangdong Academy of Medical Sciences, 510260 Guangzhou, Guangdong, China; ^4^Department of Epidemiology, Guangdong Cardiovascular Institute, Guangdong Provincial People's Hospital, Guangdong Academy of Medical Sciences, 510260 Guangzhou, Guangdong, China

**Keywords:** ventricular septal defect, perventricular device closure, conventional surgical repair, meta-analysis, aortic regurgitation

## Abstract

**Background:**

This systematic review and meta-analysis aimed at comparing the midterm outcomes of perventricular device closure (PDC) with conventional surgical repair (CSR) for VSD.

**Methods:**

PubMed, Cochrane Library, and Web of Science databases were searched from January 1, 2005, to October 15, 2020, for English or Chinese language studies comparing outcomes of PDC with CSR for VSD. The midterm results were assessed as a primary outcome. A systematic review and meta-analysis was performed under the frequentist frame with risk ratio (RR) and 95% confidence interval (CI).

**Results:**

A total of 4381 patients (PDC = 2016, CSR = 2365) from 15 studies were included. The pooled estimates of success rate favored the CSR compared with the PDC (RR, 0.97; 95% CI, 0.96 to 0.99; *p* = 0.001). No significant differences in minor complications or severe complications were found between the PDC and CSR (RR, 0.79; 95% CI, 0.50 to 1.23; *p* = 0.29; RR, 1.43; 95% CI, 0.74 to 2.75; *p* = 0.29). The pooled estimates of residual shunts favored the PDC compared with the CSR (RR, 9.07; 95% CI, 4.77 to 17.24; *p *< 0.001), the pooled estimates of aortic regurgitation favored the CSR compared with the PDC (RR, 1.59; 95% CI, 1.05 to 2.39; *p* = 0.03).

**Conclusions:**

PDC is a safe and effective procedure with less surgical injury and shorter perioperative hospital stay. However, aortic regurgitation is a concern during follow-up.

## 1. Introduction

Ventricular septal defect (VSD) is one of the most common congenital heart malformations, accounting for approximately 20% of congenital heart defects [1]. Conventional surgical repair (CSR) is the standard treatment for most ventricular septal defects. Current CSR results of ventricular septal defect are favourable, with low mortality rates and acceptable long-term follow-up outcomes [2, 3, 4]. However, median sternotomy and cardiopulmonary bypass (CPB), which are required during surgical repair, have some disadvantages, e.g., surgical scars, longer postoperative hospital stay, and sternal deformity [5, 6]. To avoid the shortcomings of CSR, Lock j *et al*. [7] introduced transcatheter closure technology into the treatment of ventricular septal defects in 1988. After decades of development, percutaneous catheter closure of ventricular septal defects has been proven to be a valuable option for perimembranous and muscular ventricular septal defects. This technique can be used to perform the closure of intracardiac shunts with the aid of cardiac X-ray or echocardiography [8, 9, 10]. However, the application of percutaneous catheter closure was limited to specific subtypes of VSD and the size of infantile vessels. With the development of occlusion technology, some studies suggested a technique using occluders for ventricular septal defects under direct cardiac vision [11]. Liu *et al*. [12] reported preliminary results of this minimally invasive technique applied to various types of ventricular septal defects. However, this technique still has serious complications, such as aortic regurgitation and complete atrioventricular block. In addition, this technology has been widely used in China, but it has not been popularized all over the world.

This review aimed at comparing the midterm outcomes between perventricular device closure (PDC) and CSR in the treatment of VSD.

## 2. Materials and Methods

This study was conducted according to PRISMA (preferred reporting items for systematic reviews and meta-analyses) guidelines [13].

### 2.1 Data Sources and Search Strategy

We searched PubMed, EMBASE, Web of Science and CNKI from January 1, 2005 to October 15, 2020. The keywords were: heart septal defects, ventricular; ventricular septal defect; closure; perventricular. The detailed search strategy is shown in **Supplementary Fig. 1**.

### 2.2 Selection Criteria, Data Extraction, and Quality Assessment

Two investigators independently reviewed all search records, and no disagreements emerged. Specific inclusion criteria were: (1) direct comparative studies of PDC and CSR; (2) isolated congenital VSD in surgical patients (Patient selection criteria: (i) congenital VSD (ii) aortic valve prolapse but no more than mild regurgitation of the aortic valve was present, (iii) no more than moderate regurgitation of the atrioventricular valve was present and (iv) no other malformations which needed repair except for a patent foramen ovale (≤5 mm)); (3) the following data were involved in the articles: surgical success rate (with or without reason for surgical failure), length of intensive care unit (ICU) and hospital stay, post-operative (within 30 days) complications (including residual shunts, arrhythmia, transfusion and other complications such as death); (4) mean follow-up ≥six months; and (5) required data including residual shunts, conduction blocks, valvular regurgitation during follow-up. Exclusion criteria were duplicate publications, study sample <30 patients, and ambiguous end-point measures.

Data extraction was performed using Microsoft Excel 2013 (Microsoft, Redmond, WA, USA). Standard data abstraction forms were created to collect data of interest. Two independent investigators extracted the following data including first author, publication year, sample, mean age, mean weight, VSD size, surgical closure procedures, mean follow-up time, and post-operative complications including minor and major complications. The major complications were defined as death, reintervention for residual shunts or valvular regurgitation, conduction block requiring pacemaker implantation, other reasons for the need for reoperation. The minor complications were defined as non-intervention complications, such as transfusion, pulmonary infection, and pneumothorax.

The quality assessment of included studies was performed using the Newcastle Ottawa scale (NOS) [14]. According to the NOS, each study was judged on three broad perspectives: the selection of the study group; the comparability of the groups; and the ascertainment outcome of interest. According to the NOS, a study can be awarded a maximum of one star for each numbered item in the Selection and Outcome categories. A maximum of two stars can be given for Comparability.

### 2.3 Procedure

**Fig. 1.**
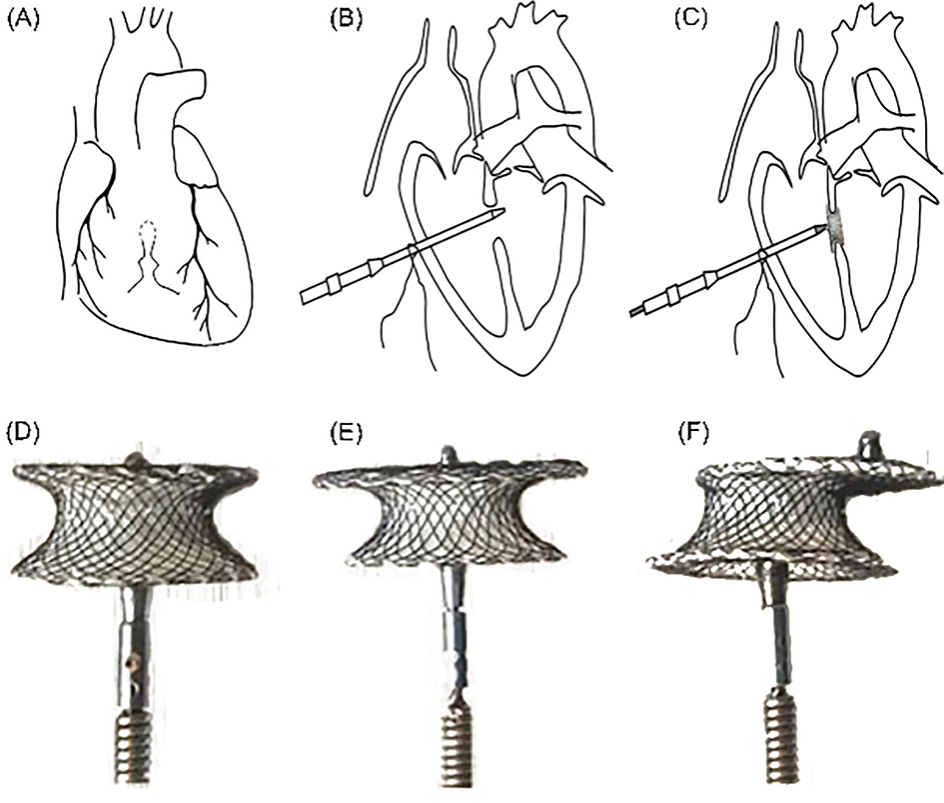
**Schematic illustrating the perventricular device closure of VSD and the type of occluder**. (A) Under the guidance of TEE, a suitable puncture site was selected in the right ventricle, and a mattress suture with a gasket was placed. (B) Position the conveying sheath. (C) The occluder was placed, TEE repeatedly evaluated that the occluder was in good position, and finally release the occluder. (D) Concentric occluder. (E) Small-waist-big-sided occluder. (F) Eccentric occluder.

There are various methods of performing PDC in different centers. We introduced a general approach, which is mentioned in much of the literature and has also achieved good results. PDC was performed under general anesthesia, tracheal intubation and TEE guidance. Before the procedure, TEE was used to re-evaluate the position, shape and size of the VSD and adjacent structures, especially its relationship with the aortic valve, to help select the appropriate device and delivery system. A small incision was made at the lower sternum, subxiphoid or left intercostal space. A small portion of the pericardium was incised to expose the free wall of the right ventricle. The bag was then sutured and then inserted into the conveying sheath; the occlude was then positioned and released in the appropriate position. TEE was used in all cases during the entire procedure to guide and check the device position, to evaluate tricuspid and aortic valve insufficiency and leaflet motion, and to detect residual shunts (Fig. 1A,B,C). The selection of the occluder should be individualized according to the specific anatomy of each patient. The location, size, morphology of the VSD, thickness of the interventricular septum at the corresponding site, and relationship of the defect to surrounding vital structures need to be considered (Fig. 1D,E,F). Conventional surgical repair was conducted through a median sternotomy approach under CPB.

### 2.4 Statistical Analysis

Review Manager 5.4 software (Cochrane Collaboration, Copenhagen, Denmark), developed by Cochrane Collaboration, was used for statistical analysis. Pooled risk ratio (RR) was reported as 95% CI, and *p *< 0.05 was considered statistically significant. The Cochran’s Q test and $I^{2}$ test were performed to judge the heterogeneity of these studies in the meta-analysis. Heterogeneity was considered significant at *p *< 0.1 for the Q statistic. $I^{2}$ values less than 50% indicated low heterogeneity, values between 50% and 75% indicated moderate heterogeneity, and $I^{2}$ greater than 75% was considered high heterogeneity. The fixed-effects model analyzed the results without significant heterogeneity, and the random-effects model analyzed the results with significant heterogeneity. Sensitivity analysis was performed by successively excluding low-quality studies to evaluate the stability of the results. Potential publication bias was evaluated by constructing a funnel plot. The plot was estimated visually, and the asymmetric funnel plot suggested possible publication bias.

## 3. Results

**Fig. 2.**
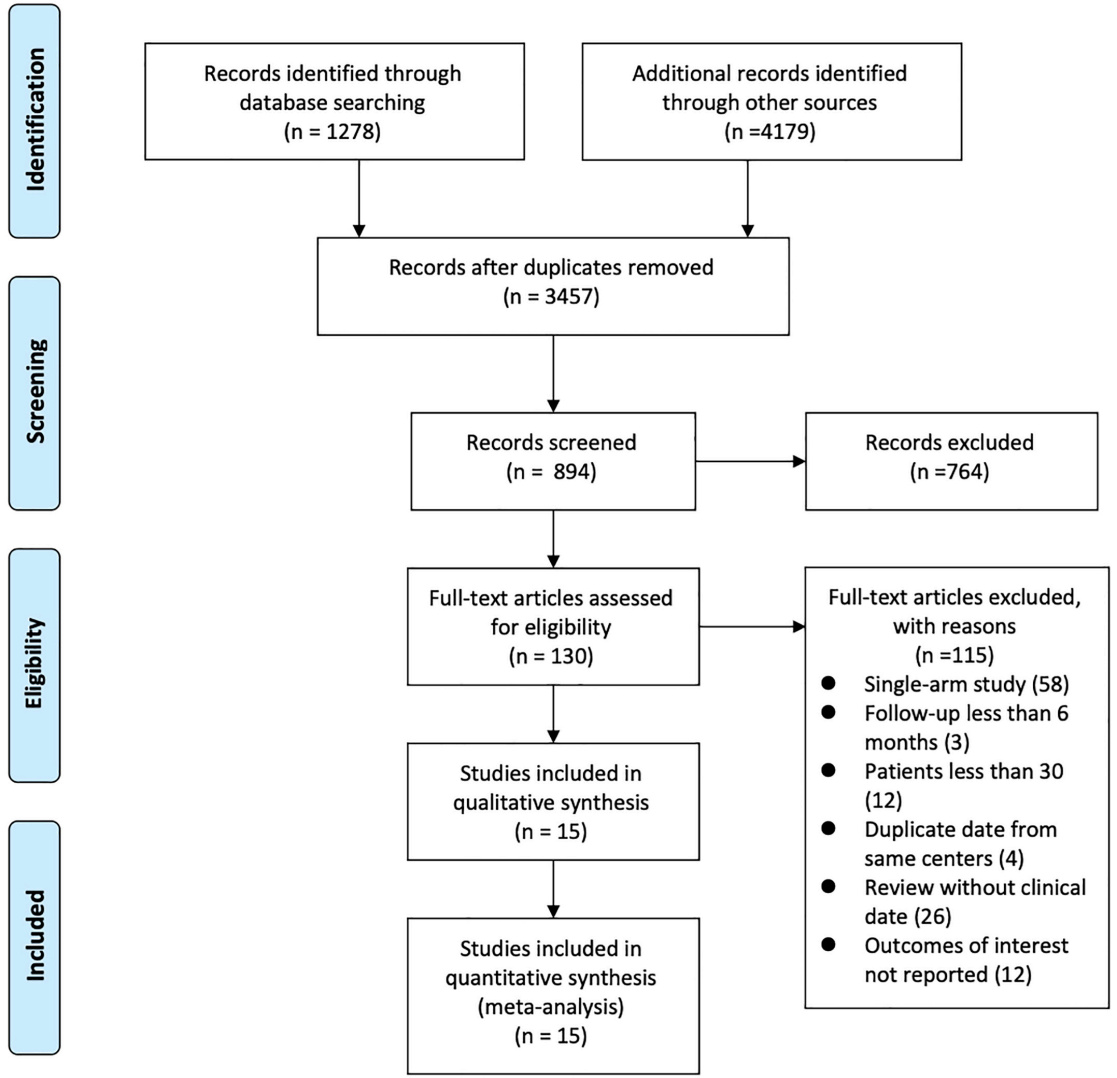
**Flow diagram of included studies**.

The literature search yielded 5457 published articles. Further screening and exclusion reduced these to 15 articles (including 4381 patients) which were used for the statistical analysis [15, 16, 17, 18, 19, 20, 21, 22, 23, 24, 25, 26, 27, 28, 29]. Among them, three papers [23, 26, 28] were 3-arm studies comparing the efficacy of transcatheter closure, perventricular closure, and surgery, and the rest were direct comparisons. Of the 15 studies evaluated, most were observational and retrospective in design, and only one study [25] was a randomized controlled trial. The PRISMA flow diagram with the description of the study selection process is presented in Fig. 2.

### 3.1 Preoperative Patient Characteristics.

There was no difference in the preoperative clinical characteristics between the two groups. The mean ages were 3.15 ± 2.88 years in the PDC group and 3.47 ± 2.78 years in the CSR group, respectively. The mean body weights were 14.11 ± 5.95 kg in the PDC group versus 14.09 ± 5.49 kg in the CSR group, respectively. The percentage of males was 56.5% in the PDC group vs. 53.1% in the CSR group, and the ventricular septal defect size was 5.54 ± 0.86 vs. 6.21 ± 1.02. Detailed characteristics were shown in Table 1 (Ref. [15, 16, 17, 18, 19, 20, 21, 22, 23, 24, 25, 26, 27, 28, 29]).

**Table 1.** **Studies and patients’ baseline characteristics**.

Author (year)	Total PT (n)		Age* (y)		Female (n)		Weight* (kg)		VSD Size* (mm)		Hospital Stay* (d)		ICU Stay * (h)	
	PDC	CSR	PDC	CSR	PDC	CSR	PDC	CSR	PDC	CSR	PDC	CSR	PDC	CSR
Chen (2013) [18]	89	58	13.3	13.7	…	…	34.2	34.6	…	…	6.1	8	10.5	13.7
Yang (2015) [23]	78	210	7.9	8.5	31	58	24.1	21.7	4.5	4.7	8.1	12.4	31.2	52.8
Chen, Qiang (2019) [27]	72	63	1.3	1.1	37	33	8.3	8.2	4.2	5.2	2.2	7.1	6.7	14.3
Chen, Qin (2019) [28]	63	72	2.7	2.7	31	37	19.3	18.8	4.4	4.3	2.5	6.6	6.8	14.2
Fang (2018) [26]	90	86	1.6	1.4	42	40	10.1	9.5	5.3	5.9	4.2	8.5	13.7	22.6
Hu (2014) [19]	161	302	3.7	3.82	77	146	16.7	15.6	6.95	6.81	5.1	5.75	6.66	14.15
Hu (2015) [21]	33	96	5.3	4.4	18	54	18.1	16.4	5.1	4.2	5.4	8.2	29	46.9
Liao (2020) [29]	103	336	4.55	2.86	62	247	18.42	12.56	4.84	6.27	5.4	8.6	22.27	48.45
Ma (2019) [15]	30	32	5.5	7.7	16	11	18.0	23.7	4.9	10.1	4.3	7	…	…
Voitov (2017) [25]	320	320	2.87	3.02	157	138	13.9	14.5	5.3	6.2	7.64	16.71	16.4	38.2
Wang (2012) [16]	116	104	6.2	7.2	62	53	18.8	20.5	…	…	7.7	9.8	…	…
Xing (2015) [22]	458	283	0.95	0.86	217	131	9.82	8.56	5.21	6.83	3.82	8.55	…	…
Xu (2012) [17]	89	97	0.73	0.51	47	49	9.8	7.7	5.1	6.4	9.1	14.3	19.2	60
Zhang (2015) [24]	265	265	1.17	1.2	129	127	8.94	8.58	7.05	7.24	5.24	7.81	15.7	32.03
Zhu (2014) [20]	49	41	5.82	4.36	…	…	20.28	16.8	5.03	6.03	7.89	10.8	23.28	66.72
Total n/N (± SD)	2016	2365	3.15 ± 2.92	3.48 ± 2.81	1000/1878	1284/2266	14.25 ± 6.01	14.13 ± 5.54	5.56 ± 1.84	6.23 ± 0.99	5.56 ± 1.84	9.74 ± 3.42	15.64 ± 6.51	36.00 ± 15.63

Pt, patients; n, number; PDC, perventricular device closure; CSR, conventional surgical repair; VSD, ventricular septal defect; * means value; SD, standard deviation; kg, kilograms; mm, millimeter; d, days; y, years; h, hours.

### 3.2 Early Outcome

**Fig. 3.**
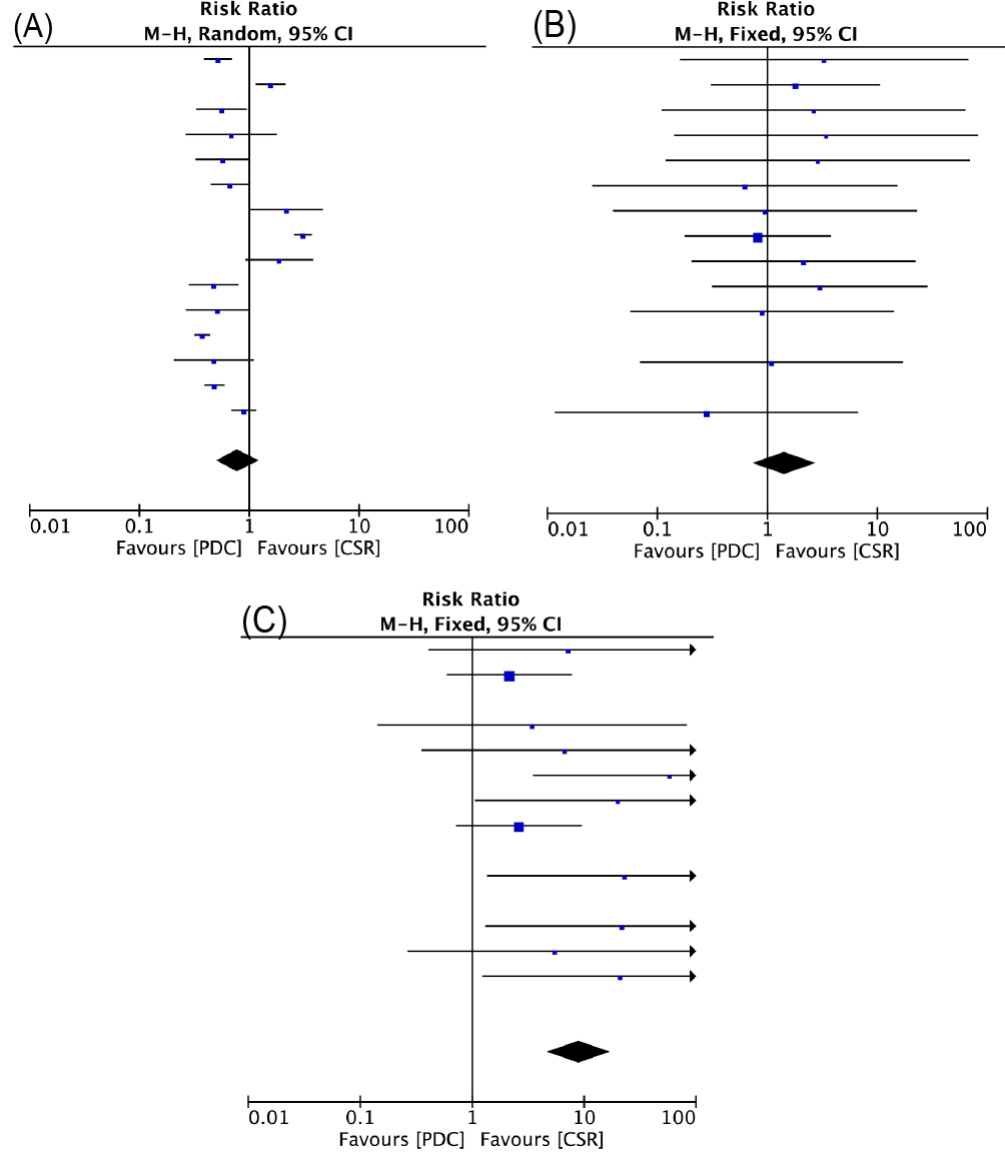
**Forrest plots of comparison (Early outcome): failure rate (A), minor complications (B), severe complications (C)**.

The CSR group required longer times of hospitalization and ICU stay (*p *< 0.001). Pooled estimates of operative success rate favored the CSR compared with the PDC (96.3% vs. 99.5%; RR, 9.07; 95% CI, 4.77 to 17.24; *p* = 0.001). No significant difference was found in minor complications (RR, 0.79; 95% CI, 0.50 to 1.23; *p* = 0.29) and major complications (RR, 1.43; 95% CI, 0.74 to 2.75; *p* = 0.29) (Fig. 3).

### 3.3 Midterm Outcome

**Fig. 4.**
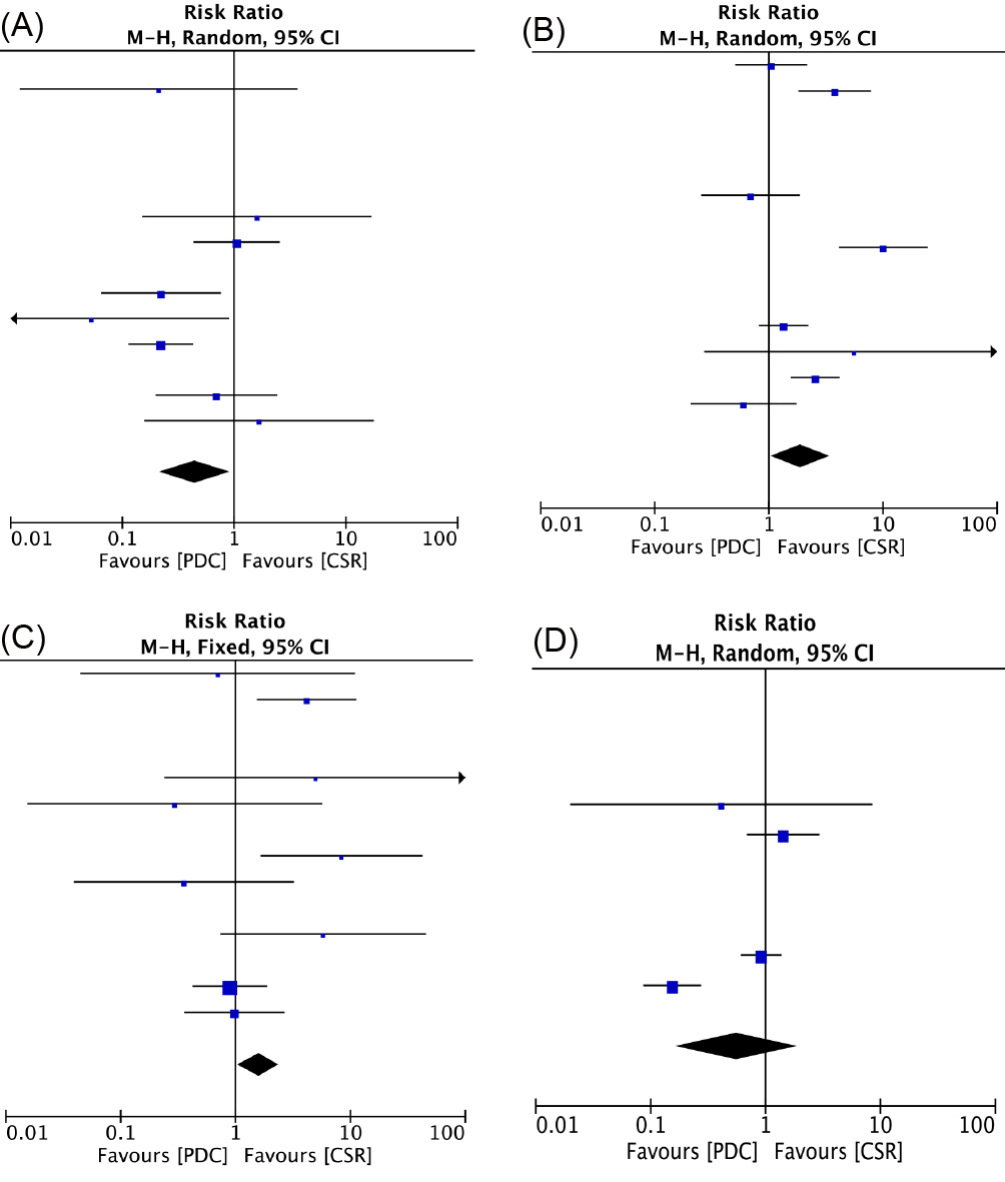
**Forrest plots of comparison (Midterm outcome): residual shunts (A), tricuspid regurgitation (B), aortic regurgitation (C), right bundle branch block (D)**.

The median follow-up duration was 20 (Range, 12 to 30) months. During the follow-up, there was no late deaths in either group. All the publications reported residual shunts during follow-up, and the pooled estimates of the incidence of residual shunts favored the device groups compared with the conventional group (RR, 0.45; 95% CI, 0.22 to 0.93; *p* = 0.03). Fourteen studies (14/15) reported tricuspid regurgitation and aortic regurgitation [15, 17, 18, 19, 20, 21, 22, 23, 24, 25, 26, 27, 28, 29], pooled estimates of tricuspid regurgitation favored the PDC compared with the CSR (RR, 1.9; 95% CI, 1.04 to 3.45; *p* = 0.04). The pooled estimates of aortic regurgitation favored the CSR compared with the PDC (RR, 1.59; 95% CI, 1.05 to 2.39; *p* = 0.03). Complete atrioventricular block and Mobitz II block have not been reported. Eleven studies (11/15) reported right bundle branch blocks [15, 16, 18, 19, 21, 22, 24, 25, 26, 27, 28], in which the incidence between the two groups was not significantly different (RR, 0.56; 95% CI, 0.17 to 1.87; *p* = 0.35) (Fig. 4).

### 3.4 Risk of Bias

**Fig. 5.**
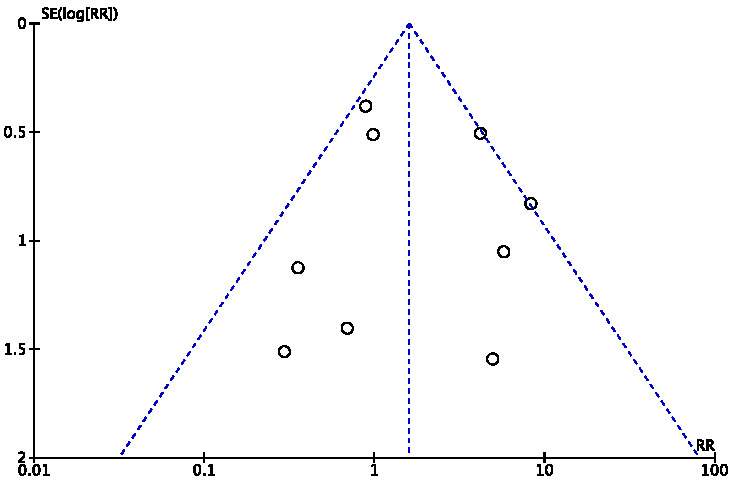
**Funnel plot of aortic regurgitation**.

**Fig. 6.**
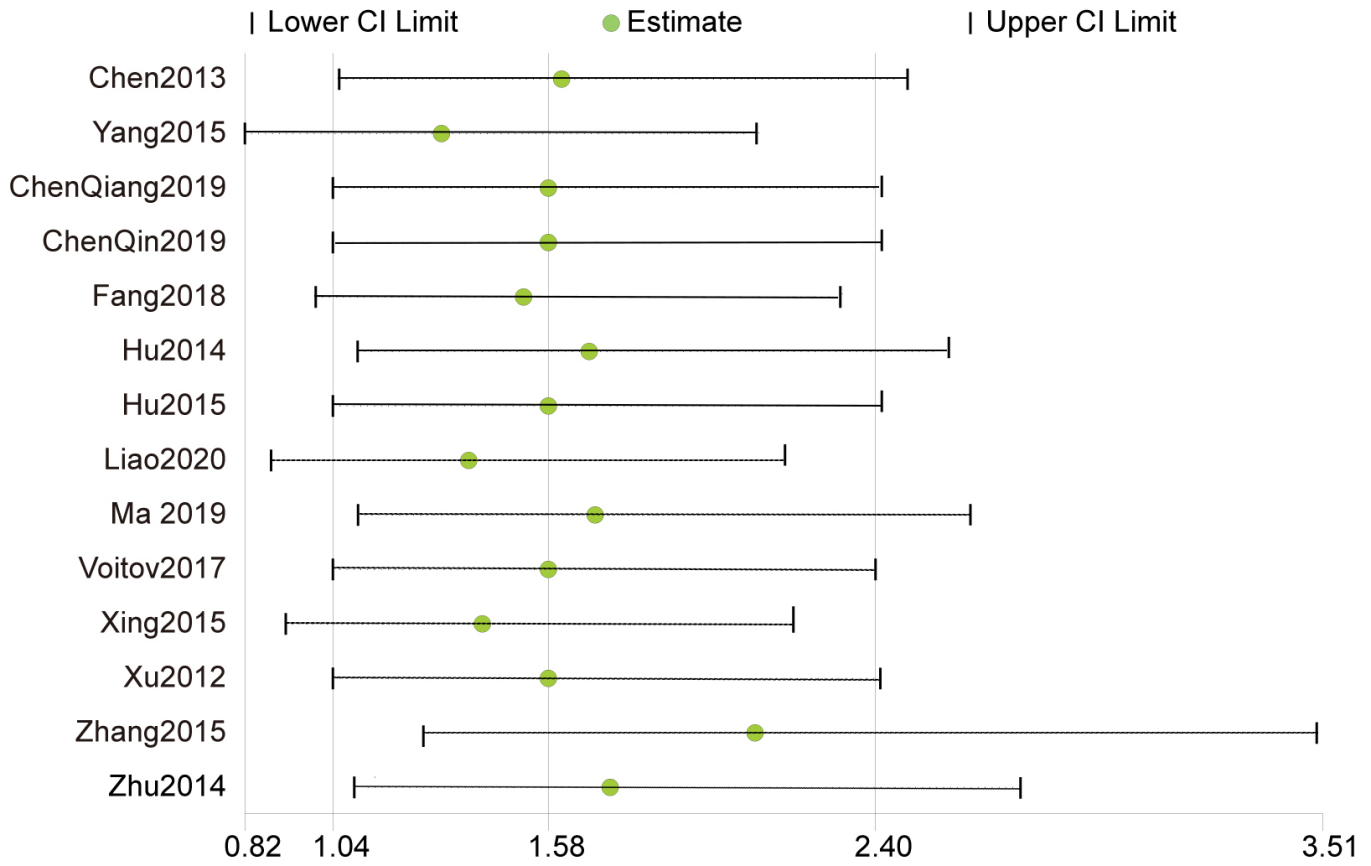
**Sensitivity analysis of aortic regurgitation**.

The details regarding quality assessment and risk of bias were shown in Table 2 (Ref. [15, 16, 17, 18, 19, 20, 21, 22, 23, 24, 25, 26, 27, 28, 29]). Although the research quality of studies differed from one another, a lack of randomization and double-blind remained major obstacles. After excluding each individual study, the sensitivity analysis did not show any difference between the two groups. Fig. 5 showed the funnel plot of aortic regurgitation, without publication bias. Fig. 6 showed the sensitivity analysis of aortic regurgitation.

**Table 2.** **Risk-of-bias assessments**.

Author (year)	Selection				Comparability	Outcome			Total scores
	Is the case definition adequate?	Representativeness of the cases	Selection of Controls	Definition of Controls	Comparability of cases and controls on the basis of the design or analysis	Ascertainment of exposure	Same method of ascertainment for cases and controls	Non- Response rate	
Chen (2013) [18]	★	★	☆	★	★☆	★	★	★	7
Yang (2015) [23]	★	★	☆	★	★☆	★	★	★	7
Chen, Qiang (2019) [27]	★	★	☆	★	★ ★	★	★	★	8
Chen, Qin (2019) [28]	★	★	☆	★	★☆	★	★	★	7
Fang (2018) [26]	★	★	☆	★	★ ★	★	★	★	8
Hu (2014) [19]	★	★	☆	★	★☆	★	★	★	7
Hu (2015) [21]	★	★	☆	★	★☆	★	★	★	7
Liao (2020) [29]	★	★	☆	★	★ ★	★	★	★	8
Ma (2019) [15]	★	★	☆	☆	★☆	★	★	★	6
Voitov (2017) [25]	★	★	☆	★	★ ★	★	★	★	8
Wang (2012) [16]	★	☆	☆	★	★☆	★	★	★	6
Xing (2015) [22]	★	★	☆	★	★☆	★	★	★	7
Xu (2012) [17]	★	★	☆	★	☆☆	★	★	★	6
Zhang (2015) [24]	★	★	☆	★	★ ★	★	★	★	8
Zhu (2014) [20]	★	★	☆	★	★☆	★	★	★	8

★, yes; ☆, no.

## 4. Discussion

The main findings of our meta-analysis are: (1) PDC is safe, and is associated with a shorter hospital stay compared to CSR; (2) PDC is comparable to CSR in terms of major and minor complications; and (3) PDC was superior in terms of the incidence of residual shunts, but was associated with a higher risk of AR during midterm follow-up.

### 4.1 Early Outcome

More than 95% procedure success rates were achieved in the PDC group, but was slightly lower than that of the CSR group. Liu *et al*. [12] also reported comparable success rates in their study. An additional open surgical procedure was required in the PDC group due to recurrent aortic regurgitation, significant residual shunts, and a high degree of atrioventricular block.

There was no significant difference in the rates of major or minor complications between the two groups at discharge. Liu *et al*. [12] reported no difference between PDC and CSR in terms of residual shunts, complete AVB, RBBB, valvular regurgitation, incision infection, and pericardial effusion. Voitov *et al*. [25] also reported similar results. Zhou *et al*. [30] performed a meta-analysis of PDC versus CSR. There was no difference between the two methods in terms of residual shunts and valve insufficiency, but the risk of arrhythmia was lower in PDC [30]. Liu *et al*. [12] reported the incidence of postoperative lung injury is higher in CSR, while PDC is superior in the recovery of postoperative respiratory function.

### 4.2 Midterm Outcome

Few meta-analyses have focused on midterm outcome. Residual shunts, valvular regurgitation, and conduction blocks after follow-up greater than 6 months were analyzed in this study.

The presence of residual shunts may cause hemolysis and affect the occluder stability [30]. Some studies also reported a higher residual shunt rate in CSR compared with PDC during follow-up [22, 25]. Postoperative residual shunt rates were high, but eight studies (8/15) [16, 20, 21, 22, 23, 24, 25, 29], illustrated the high probability of spontaneous closure of residual shunts during follow-up. Maartje Schipper *et al*. [31] reported that 71% of postoperative residual shunts will all close spontaneously and that the final spontaneous closure rate is high during the last follow-up. In their report, only one case required reoperation for a large residual shunt during follow-up [21], while the remaining residual shunts were small, without progression during follow-up.

Tricuspid regurgitation was all mild and stable at the last follow up. Rahmath *et al*. [32] suggested that impingement of the device on the septal leaflet of the tricuspid valve, and rarely, rupture of the chordae tendineae, are possible etiologies for this regurgitation. Wang and colleagues [33] suggested that the presence of an anomalous origin of the tricuspid main chordae from the perimembranous ventricular septal defect seen on transthoracic echocardiography prior to the intervention is an important exclusion criterion [33]. Our study shows that the risk of tricuspid regurgitation in the PDC group is lower than that in the CSR group. Xing *et al*. [22] also suggested that PDC has a higher probability for developing minor tricuspid regurgitation than in CSR. However, Voitov *et al*. [25] reported that the risk of tricuspid regurgitation during follow-up was not significantly different between the two groups.

The results of the analysis for aortic regurgitation showed that the risk of aortic regurgitation was significantly higher in the PDC group than in the CSR, although they are mild and stable. However, Liao *et al*. [29] suggested, no significant difference in the rates of aortic regurgitation between the two groups. We believe that the more aortic regurgitation seen with PDC may be related to the following reasons: (1) The close distance between the occluder and the aortic valve might cause inevitable damage by the occluder to the aortic valve, (2) Inappropriate size of the occlude may predispose to injury in the aortic annulus and subvalvular apparatus, and (3) Intracardiac operation without direct vision may damage the aortic valve, resulting in significant aortic regurgitation.

Late atrioventricular block is also a concern in ventricular septal defect closure, however late complete atrioventricular block (CAVB) and conduction block beyond degree II were not present in our included publications. Zhang *et al*. [24] suggested the reasons for not presenting with CAVB were primarily: (1) The improvement of the delivery system and occluder technology; (2) An experienced cardiovascular surgeon can reduce the damage to the bundle branch conduction system; (3) Choosing a suitable occluder.

After reviewing the included publications, only four publications reported conduction block [20, 22, 23, 25]. CSR had a greater risk of incomplete right bundle branch block than PDC. Similar finding was reported by Xu *et al*. [17], who suggested that the right ventricular incision and its repair may damage the right side of the septum, and the right bundle branch. Pederson and colleagues [34] studied the long-term effects of right bundle branch block on left ventricular function after septal myectomy. They noted that right bundle branch block did not appear to affect systolic ventricular function but might be associated with diastolic dysfunction at long-term follow-up. Zhang *et al*. [24] reported that incomplete left bundle branch block was more common in the PDC group, in which the left bundle branches might have been damaged during guidewire advancement or occluder deployment. Right bundle branch block after CSR remained and warranted long-term evaluation.

### 4.3 Limitation

The main limitation of this study is the fact that most of the studies included in this systematic review are retrospective, and most of the researchers are Chinese. The reliability of the conclusions of our meta-analysis was subjected to confounding factors and selection bias. The comorbidities and follow-up data are based on heterogeneous data and should be treated with considerable caution. Personal and institutional experience is essential for surgical repair. Although our analysis showed no significant reporting bias, this bias remains possible. More favorable results reported by large centers might not be representative of all institutions. Because the timeframe of the study period was rather long, advances in management and operational strategies may have been a confounding factor, limiting our qualitative and quantitative analyses. In addition, most of the patients in this study are from China, which also resulted in the older age of our patients.

## 5. Conclusions

For selected patients with ventricular septal defects, PDC is a safe approach, with less surgical injury and shorter perioperative hospital stay. Severe complications during follow-up were comparable in both groups, while PDC showed a lower incidence of residual shunts. However, the risk of developing aortic regurgitation after treatment with PDC is greater and requires long-term follow-up.

## Supplementary Material

Supplementary material associated with this article can be found, in the online version, at https://doi.org/10.31083/j.rcm2308262.
