# Supplementary material for: Midterm Results Comparing Perventricular Device Closure with Surgical Repair for Isolated Congenital Ventricular Septal Defects: A Systematic Review and Meta-Analysis
Source: Rev Cardiovasc Med. 2022 Jul 20;23(8):262. doi: 10.31083/j.rcm2308262 (PMC11266945; doi:10.31083/j.rcm2308262)
Supplement: Supplementary file 1 [file 2153-8174-23-8-262-s1.docx]

Supplementary Fig. 1.
